# Recent Advances in Vaccine Development for Flaviviruses and Alphaviruses

**DOI:** 10.3390/vaccines13080808

**Published:** 2025-07-30

**Authors:** Young Chan Kim, Arturo Reyes-Sandoval

**Affiliations:** 1 Oxford Vaccine Group, Department of Paediatrics, University of Oxford, Oxford OX3 7LE, UK; 2 Centre for Human Genetics, Division of Structural Biology, University of Oxford, Oxford OX3 7BN, UK; 3 Instituto Politécnico Nacional (IPN), Av. Luis Enrique Erro s/n. Unidad Adolfo López Mateos, Mexico City 07738, Mexico; arturoreyess@ipn.mx

Mosquito-borne viruses such as dengue (DENV), yellow fever (YFV), Zika (ZIKV), and chikungunya (CHIKV) have re-emerged in recent decades, affecting millions of people worldwide [1,2,3]. These flaviviruses and alphaviruses can be classified into a broader category of arboviruses, which cause significant disease burdens and public health concerns. Vaccine development against arboviruses has experienced swift progress after the sudden re-emergence of cases of DENV, CHIKV, YFV, and ZIKV in the last two decades [1,2,3,4,5,6,7,8]. The 21st century has heralded a new era in vaccinology, driven by recombinant genetic technologies that now enable unprecedented speed in vaccine development, which has been clearly demonstrated by the rapid response to the COVID-19 pandemic [9]. A broad range of vaccine platforms, including both classical and new approaches, such as inactivated and attenuated, protein subunits, virus-like particles (VLPs), viral vectors, and nucleic acids (DNA and mRNA), is currently being tested in preclinical studies and in clinical trials [10,11,12].

A recent milestone in vaccine development for alphaviruses is the FDA’s approval of two chikungunya virus (CHIKV) vaccines—IXCHIQ, a single-dose live-attenuated vaccine (approved November 2023), and VIMKUNYA, a recombinant virus-like particle (VLP) adsorbed to aluminum hydroxide as an adjuvant (approved February 2025) [13,14]. Despite this progress, there is a continuing need for next-generation vaccines that can overcome limited supplies of inactivated or VLP vaccines and the contraindications that may restrict live-attenuated vaccines in pregnant or immunocompromised populations [15,16,17,18].

Building on the success of the first Special Issue of *Vaccines*, entitled “Vaccines Against Flaviviruses and Alphaviruses: Recent Advances and Future Challenges”, this second edition of the Special Issue will feature the latest developments in relation to vaccines against flaviviruses and alphaviruses from antigen design to clinical testing [19]. Although the overall progress against many arboviruses remains slow, a diverse pipeline of vaccine candidates spanning both classical and next-generation platforms is now in clinical trials and could lead to the future licensure of vaccines targeting these medically important emerging arboviruses.

## References

[1] Global Arbovirus Initiative. https://www.who.int/initiatives/global-arbovirus-initiative.

[2] ARBO Bulletins—PAHO/WHO|Pan American Health Organization. https://www.paho.org/en/arbo-portal/arbo-bulletins.

[3] Weaver S.C., Charlier C., Vasilakis N., Lecuit M. (2018). Zika, Chikungunya, and Other Emerging Vector-Borne Viral Diseases. Annu. Rev. Med..

[4] Girard M., Nelson C.B., Picot V., Gubler D.J. (2020). Arboviruses: A Global Public Health Threat. Vaccine.

[5] Fauci A.S., Morens D.M. (2016). Zika Virus in the Americas—Yet Another Arbovirus Threat. N. Engl. J. Med..

[6] Weaver S.C., Lecuit M. (2015). Chikungunya Virus and the Global Spread of a Mosquito-Borne Disease. N. Engl. J. Med..

[7] Simmons C.P., Farrar J.J., Van Vinh Chau N., Wills B. (2012). Current Concepts: Dengue. N. Engl. J. Med..

[8] Bhatt S., Gething P.W., Brady O.J., Messina J.P., Farlow A.W., Moyes C.L., Drake J.M., Brownstein J.S., Hoen A.G., Sankoh O. (2013). The Global Distribution and Burden of Dengue. Nature.

[9] Kim Y.C., Dema B., Reyes-Sandoval A. (2020). COVID-19 Vaccines: Breaking Record Times to First-in-Human Trials. NPJ Vaccines.

[10] Pollard A.J., Bijker E.M. (2021). A Guide to Vaccinology: From Basic Principles to New Developments. Nat. Rev. Immunol..

[11] van Riel D., de Wit E. (2020). Next-Generation Vaccine Platforms for COVID-19. Nat. Mater..

[12] Pereira C.A.d.M., Mendes R.P.G., da Silva P.G., Chaves E.J.F., Pena L.J. (2025). Vaccines Against Urban Epidemic Arboviruses: The State of the Art. Viruses.

[13] Commissioner of Food and Drugs FDA Approves First Vaccine to Prevent Disease Caused by Chikungunya Virus. https://www.fda.gov/news-events/press-announcements/fda-approves-first-vaccine-prevent-disease-caused-chikungunya-virus.

[14] Center for Biologics Evaluation and Research (CBER) (2025). VIMKUNYA.

[15] Sutter R.W., Cochi S.L. (2019). Inactivated Poliovirus Vaccine Supply Shortage: Is There Light at the End of the Tunnel?. J. Infect. Dis..

[16] CDC Contraindications and Precautions. https://www.cdc.gov/vaccines/hcp/imz-best-practices/contraindications-precautions.html.

[17] Opri R., Zanoni G., Caffarelli C., Bottau P., Caimmi S., Crisafulli G., Franceschini F., Liotti L., Saretta F., Vernich M. (2018). True and False Contraindications to Vaccines. Allergol. Immunopathol..

[18] Center for Biologics Evaluation and Research (CBER) (2025). Vaccines Licensed for Use in the United States.

[19] Kim Y.C., Reyes-Sandoval A. (2023). Recent Developments in Vaccines against Flaviviruses and Alphaviruses. Vaccines.

